# Integrated systems biology analysis of KSHV latent infection reveals viral induction and reliance on peroxisome mediated lipid metabolism

**DOI:** 10.1371/journal.ppat.1006256

**Published:** 2017-03-03

**Authors:** Zoi E. Sychev, Alex Hu, Terri A. DiMaio, Anthony Gitter, Nathan D. Camp, William S. Noble, Alejandro Wolf-Yadlin, Michael Lagunoff

**Affiliations:** 1 Molecular and Cellular Biology Program, University of Washington, Seattle, Washington, United States of America; 2 Department of Microbiology, University of Washington, Seattle, Washington, United States of America; 3 Department of Genome Science, University of Washington, Seattle, Washington, United States of America; 4 Department of Biostatistics and Medical Informatics, University of Wisconsin-Madison and Morgridge Institute for Research, Madison, Wisconsin, USA; University of North Carolina at Chapel Hill, UNITED STATES

## Abstract

Kaposi’s Sarcoma associated Herpesvirus (KSHV), an oncogenic, human gamma-herpesvirus, is the etiological agent of Kaposi’s Sarcoma the most common tumor of AIDS patients world-wide. KSHV is predominantly latent in the main KS tumor cell, the spindle cell, a cell of endothelial origin. KSHV modulates numerous host cell-signaling pathways to activate endothelial cells including major metabolic pathways involved in lipid metabolism. To identify the underlying cellular mechanisms of KSHV alteration of host signaling and endothelial cell activation, we identified changes in the host proteome, phosphoproteome and transcriptome landscape following KSHV infection of endothelial cells. A Steiner forest algorithm was used to integrate the global data sets and, together with transcriptome based predicted transcription factor activity, cellular networks altered by latent KSHV were predicted. Several interesting pathways were identified, including peroxisome biogenesis. To validate the predictions, we showed that KSHV latent infection increases the number of peroxisomes per cell. Additionally, proteins involved in peroxisomal lipid metabolism of very long chain fatty acids, including ABCD3 and ACOX1, are required for the survival of latently infected cells. In summary, novel cellular pathways altered during herpesvirus latency that could not be predicted by a single systems biology platform, were identified by integrated proteomics and transcriptomics data analysis and when correlated with our metabolomics data revealed that peroxisome lipid metabolism is essential for KSHV latent infection of endothelial cells.

## Author summary

Kaposi’s Sarcoma herpesvirus (KSHV) is the etiologic agent of Kaposi’s Sarcoma, the most common tumor of AIDS patients. KSHV modulates host cell signaling and metabolism to maintain a life-long latent infection. To unravel the underlying cellular mechanisms modulated by KSHV, we used multiple global systems biology platforms to identify and integrate changes in both cellular protein expression and transcription following KSHV infection of endothelial cells, the relevant cell type for KS tumors. The analysis identified several interesting pathways including peroxisome biogenesis. Peroxisomes are small cytoplasmic organelles involved in redox reactions and lipid metabolism. KSHV latent infection increases the number of peroxisomes per cell and proteins involved in peroxisomal lipid metabolism are required for the survival of latently infected cells. In summary, through integration of multiple global systems biology analyses we were able to identify novel pathways that could not be predicted by one platform alone and found that lipid metabolism in a small cytoplasmic organelle is necessary for the survival of latent infection with a herpesvirus.

## Introduction

Viruses have evolved functions to reprogram the proteomic landscape of their host and modulate cellular signaling pathways to adjust the regulation of cellular machinery. These cellular alterations support the survival of infected cells to allow replication and spread of the virus. Many viruses rewire host cell signaling pathways to activate the host cell and to enable lytic replication, and in the case of the herpesviruses, to support long-term latent infection [1, 2]. During latency, herpesviruses are known to modulate host cell signaling pathways that lead to inhibition of apoptosis, subversion of the host immune response, and alteration in host carbon and lipid metabolism among many other pathways. Importantly, alteration of these pathways by some oncogenic gamma-herpesviruses may influence tumor formation given the optimal cellular milieu [3, 4].

Kaposi’s Sarcoma Associated Herpesvirus (KSHV), a human gamma-herpesvirus, is the etiological agent of Kaposi Sarcoma and two B-cell lymphoproliferative diseases, Primary Effusion Lymphoma (PEL) and Multicentric Castleman Disease (MCD) [5–7]. KS is the most common AIDS-associated malignancy worldwide and among the most common tumors overall in Sub-Saharan Africa [8]. KSHV is found in the main KS tumor cells, the spindle cells, which are cells of endothelial origin [9, 10]. In the KS spindle cells, KSHV is predominantly in the latent state (>90%) where only a handful of the more than 90 annotated viral genes are expressed as well as a number of viral microRNAs [11, 12]. A limited number of spindle cells (< 5%) express markers of lytic replication as well [13]. While there are limited animal models for the disease, there are well-established mammalian cell culture systems that recapitulate the latent and lytic infection rates seen in KS tumors [14–17]. We and others have successfully used these cell culture models to demonstrate that KSHV promotes angiogenesis, modulates carbon utilization and alters lipid profiles in KSHV latently infected endothelial cells [18–21]. Our previous work showed that latent KSHV infection leads to profound changes in central carbon metabolism and fatty acid (FA) synthesis and that both are required for the survival of latently infected cells indicating the importance of altered metabolism and lipid homeostasis to latent infection [19, 22]. Many of these cellular changes induced by KSHV are similar to phenotypes that commonly occur in cancer cells [3].

Several of the signaling pathways modulated by KSHV infection have been studied through traditional approaches of identifying individual host proteins or pathways predicted to play a role in the phenotype investigated. Here we are applying a more comprehensive approach where the global response of cell host in response to KSHV infection during latency at the protein and transcript levels are evaluated. Systems biology approaches can be utilized to identify important cellular networks on a cell-wide scale. In particular, advancement of recent mass spectrometry-based techniques using affinity-based phosphopeptide enrichment coupled with chemical labeling and high-resolution chromatography have been adapted to query changes in protein phosphorylation [23–25]. In addition, the assembly of large-scale, high-quality, protein-protein interaction databases provide an extensive and detailed context for interpreting proteome changes [26]. To evaluate gene expression profiles, next generation sequencing technology provides comprehensive analysis of the presence and quantity of the transcriptome. The use of transcriptomics data to predict transcription factor (TF) activity as a function of changes in mRNA provides an effective tool to link proteomics to transcriptomics data [27]. The integration of these two different data types has been successfully demonstrated in several biological systems, including glioblastoma [28], breast cancer [29], epithelial-mesenchymal transition [30], yeast salt stress response [31] and influenza virus infection [32]. These studies have provided insights and a comprehensive view of cellular networks from stimuli to gene expression/suppression.

We performed a systems-level data integration approach to identify global changes in cellular networks that are important for KSHV latent infection. To dissect cellular changes and examine the signal transduction from upstream signaling to downstream targets induced by KSHV infection, we first conducted a mass spectrometry-based proteomics and phosphoproteomics analysis, including both tyrosine and serine/threonine phosphoproteomics. We also evaluated gene expression profiles following KSHV infection using high throughput sequencing to generate global cellular transcriptomics data. Virally induced changes in both the proteome and the transcriptome were integrated using an inference algorithm to predict TF activation. A comprehensive protein-protein interaction network was used to identify predicted cellular pathways subverted by KSHV. This integrated systems biology approach identified multiple pathways altered by KSHV infection including peroxisome metabolism.

Peroxisomes have been identified as a nexus of lipid metabolism and signaling [33, 34]. While we have previously shown that FA synthesis is required for the survival of endothelial cells latently infected with KSHV, how these downstream FAs are utilized and why they are necessary have not been determined [19]. Peroxisomes have been studied in the context of infection with RNA viruses including influenza [35–40]. Interestingly, infection with influenza virus led to an increase in peroxisomes while infection with flaviviruses led to a significant decrease in peroxisomes metabolism. Our results show that KSHV latent infection of endothelial cells leads to increased numbers of peroxisomes. One major function of peroxisomes is to metabolize very long chain fatty acids (VLCFAs). Peroxisomal defects have been associated with several clinical disorders, including. Zellweger syndrome, a disease characterized by abnormal peroxisome lipid metabolism presenting with deficiency of ACOX1 function, D-bifunctional protein (D-BP) and X-linked adrenoleukodystrophy (X-ALD) [41]. Lipidomics analysis in fibroblasts cells from these patients present with abnormal lipid profiles specifically high levels of VLCFs and low levels of DHA indicating abnormal function of VLCFs breakdown and DHA synthesis [42]. ABCD3 is a peroxisomal lipid transporter of VLCFAs involved in transporting 24:6n3, the precursor of DHA [34]. After 24:6n3 is transported into the peroxisome; it gets further metabolized by ACOX1, a peroxisomal enzyme. ACOX1 synthesizes DHA by partial β-oxidation of 24:6n3 [43, 44]. In the current studies, transient knockdown of ACOX1 and ABCD3 led to cell death in the KSHV latently infected endothelial cells but not the mock-infected control. Overall, these findings validate our integrated global approach and strongly suggest that KSHV modulates peroxisomal lipid metabolism for the increased maintenance of latently infected cells.

## Results

### KSHV alters the proteome and phosphoproteome of endothelial cells during latency

**Fig 1:**
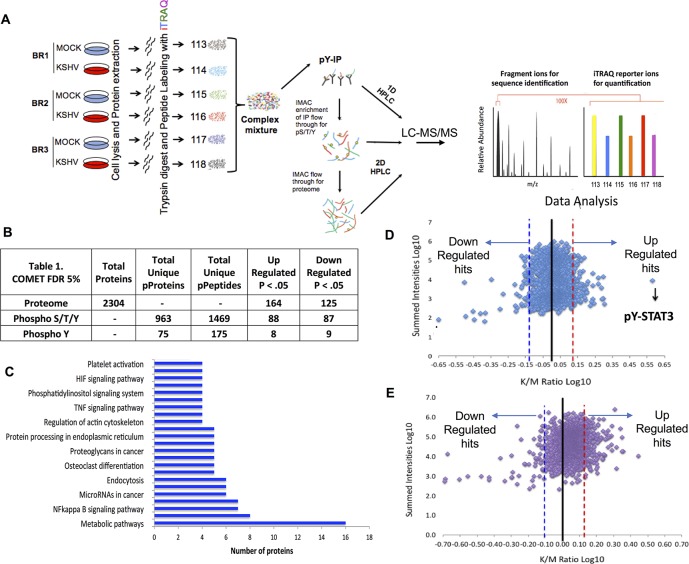
Phosphoproteome and Proteome Profiling of endothelial cells infected with KSHV. **A.)** Overview of the workflow of proteomics and phosphoproteomics sample preparation and data collection. TIME cells were infected with KSHV derived from BCBL-1 cells and harvested 48 hpi, labeled with iTRAQ and used for tyrosine phospho-proteomic analysis, global phosphoproteomic analysis, including serine and threonine phosphopeptides, and global proteomic analysis using LC-MS/MS. Biological Replicate (BR), Immunoprecipitation (IP), phosphotyrosine, phosphoserine, phosphothreonine (pY/pS/pT), 1-dimesional or 2- dimensional High Pressure Liquid Chromatography (1D HPLC, 2D HPLC) Liquid Chromatography Mass Spectrometry (LC-MS/MS). **(B.)** Table showing the number of proteins detected and the upregulated and downregulated proteins in the proteome as well as the specific phosphopeptides detected from the phosphoproteome following KSHV using Comet peptide search algorithm with a FDR 5% and p < .05. **(C.)** KEGG pathway analysis of the upregulated hits of the phospho and proteome analysis. **(D and E.)** Scatter plots demonstrating changes in relative abundance for the peptides detected in the phosphoproteome (D) and total proteome (E) following KSHV 48 hpi. The dotted lines represent the significance cut off where the points to the right of the red dotted line are up significantly upregulated hits and points to the left of the blue dotted line are significantly downregulated hits.

To quantify global signaling events modulated by KSHV, we used quantitative phosphoproteomics and proteomics to compare mock and KSHV infected endothelial cells (Fig 1A). Tert-immortalized microvascular endothelial cells (TIME) [16] were mock or KSHV infected and harvested at 48 hours- post-infection (hpi), when latency has been established. Three biological replicates were performed with separate infections performed on different days (Fig 1A). Latent infection, in greater than 90% of the cells was confirmed by immunofluorescence (IFA) assays. This approach identifies the presence of a latent protein (ORF73) and the absence of ORF59, a protein marker of lytic infection. ORF59 stained positive in less than 2% of the infected cells in all experiments. To quantify differential peptide expression levels between mock and KSHV infected cells, each sample was chemically labeled with isobaric tags for relative and absolute quantification (iTRAQ) [45] (Fig 1A). Peptide quantification was normalized prior to labeling using quantitative fluorimetric peptide assay to ensure that similar amounts of peptides were labeled across all samples. Labeled peptides from each biological sample were pooled (Fig 1A). Peptides were, then separated, sequenced and analyzed using one or two-dimensional (1D or 2-D) HPLC tandem high-resolution mass spectrometry (LC- MS/MS) for phosphotyrosine enrichment and total phosphroteome and proteome, respectively (Fig 1A).

LC-MS/MS analyses were conducted in three stages. First, low-abundance phosphotyrosine (pT) containing peptides were identified and quantified after enrichment by immunoprecipitation (IP) (Fig 1A). The IP flow-through was then used to enrich and quantify peptides containing phosphorylated serine (pS), threonine (pT) and the remaining tyrosine residues using immobilized metal affinity chromatography (IMAC). Finally, the IMAC flow-through was used to quantify total protein levels (Fig 1A). Upon data acquisition and analysis, we confirmed there was not a statistically significant difference between the mean relative abundance of peptides across the samples, indicating that the sample labeling was equally effective in each case (S1A–S1D Fig). A total of 2304 unique proteins from the proteome and 1038 unique phospho-proteins from the phosphoproteome runs were analyzed that includes phospho-tyrosine/threonine and serine. Activation of a phosphorylated residue within the same protein can vary; therefore, we analyzed individual peptides. From the phosphoproteome, we analyzed 1644 unique phospho-peptides, including 175 unique phosphotyrosine peptides that comprised 75 unique phosphotyrosine proteins (Fig 1B). The protein and peptide population distribution for the proteome and phosphoproteome, respectively, were plotted based on the sum of relative peptide intensities from the iTRAQ reporter ions from mock and KSHV infected samples versus log10 of the ratios/fold change of KSHV over mock (Fig 1D and 1E and S2 Table). Of the 1644 unique phospho-peptides identified, 192 were differentially phosphorylated, of which half were upregulated and half were down regulated (paired t-test p < .05). Phosphorylated signal transducer and activator of transcription 3 (STAT3) is the top hit of the tyrosine-phosphorylated residue from the phosphoproteome analysis (Fig 1D). Our lab has previously shown that KSHV induces persistent activation of phospho-STAT3 during latency validating the phosphoproteomic results [46]. From the upregulated hits including both phosphoproteome and proteome, there are several proteins involved in metabolism, immunity, insulin resistance, endocytosis, NFk-B signaling and others, providing potentially interesting targets for future study. From the proteome analysis, we measured 2304 unique proteins among which 289 were altered by KSHV latent infection; 164 were upregulated and 125 downregulated. This corresponds to viral induced changes in 13% of the proteins detected and 12% of the phosphorylated residues, including unique phosphotyrosine (Fig 1B). Kyoto Encyclopedia of Genes and Genomes (KEGG) pathway analysis of the phosphoproteins and proteins measured and altered during KSHV infection identified several pathways consisting of more than 4 proteins annotated [47] (Fig 1C). These pathways include metabolic processes involved in carbon and lipid metabolism as well as hypoxia inducible factor (HIF) signaling, both of which had been previously associated with KSHV latent infection, providing internal positive controls for our proteomic data [18, 22, 48]. Gene Ontology analysis also provided similar results (S2B Fig).

### KSHV alters the cellular transcriptome of endothelial cells during latency

**Fig 2:**
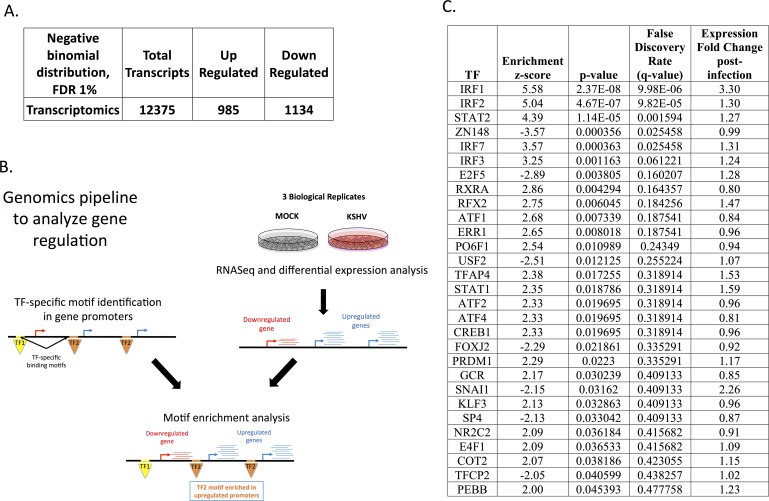
Transcription factor (TF) enrichment analysis. **(A)** Total mRNAs identified in the endothelial cells and the numbers upregulated and downregulated following infection with KSHV using a negative binomial distribution with an FDR% of 1 **(B.)** Schematic of RNAseq and TF enrichment analysis. Three biological replicates of KSHV infected endothelial cells harvested at 48 hpi for RNAseq of mRNA were analyzed. TF-specific binding motifs identified upstream of transcription start sites and differential expression p-values between mock and KSHV-infected cells from RNASeq data were used for the motif enrichment hypothesis tests. Colored arrows represent transcription start sites. Red and blue lines represent downregulated and upregulated transcripts, respectively. Triangles represent transcription factors binding upstream of transcription start site. (**C.**) List of significant TFs that are predicted to be altered by KSHV during latency with a p < .05 that were used for the Steiner forest analysis.

To identify changes at the transcriptional level, high throughput cDNA sequencing from mRNA was performed to identify genes expression differences between mock and KSHV infected endothelial cells at 48 hpi. Three separate mock and KSHV infections of TIME cells performed on different days were analyzed by high throughput sequencing of cDNA. Expression of 12,375 cellular genes in all replicates were identified. Of the genes measured, 985 cellular genes were significantly upregulated following KSHV infection of endothelial cells and 1,134 were significantly downregulated at a 1% FDR using a method based on the negative binomial distribution (Fig 2A and S3A Fig).

### Putative transcriptional regulators inferred in KSHV latently infected endothelial cells

The transcriptomic data was used to predict the activities of transcription factors based on binding motifs in the promoter regions of transcripts that are activated or repressed following latent KSHV infection. The enrichment of a motif in the promoters of genes whose expression is significantly altered implicates the motif’s associated TF as a possible regulator (Fig 2B). The software FIMO identified putative binding sites by motif presence [49], and two-sided Wilcoxon rank-sum tests [50] quantified and assigned p-values to the enrichment of those binding sites in promoters of genes significantly changed in expression after KSHV infection (Fig 2B).

FIMO was used to scan for the locations of 426 TF binding motifs, from a curated database of position-weight matrices compiled and derived from multiple experimental types, in 1000bp regions upstream of annotated transcription start sites [27]. The enrichment scores of the 261 motifs whose corresponding TF’s mRNAs were reliably detected in the RNA-seq data and the mRNA’s fold-change in expression after KSHV infection (Fig 2C). A positive enrichment z-score indicates that the motif’s putative target genes increase in expression on average, and a negative enrichment score indicates that the motif’s putative target genes decrease in expression on average. Wilcoxon rank-sum tests assigned statistical significance to the motif enrichments and found that five motifs were significantly enriched at a 5% FDR (p-value < 0.001) and twenty-four more were enriched at a less stringent cutoff of p-value < 0.05 (Fig 2C).

The motif of four of these TFs, interferon regulatory factors 1, 2, 7 (IRF1, IRF2, IRF7) and STAT2, are enriched only in upregulated promoters. However, because the motifs for these factors are similar (S3C Fig), it is not clear which of the TFs or TF complexes are actually relevant from just the motifs. The mRNA of IRF1 exhibits a 3.3-fold increase in gene expression (p-value < 0.001) as measured by our transcriptomics data, which may imply that IRF1 is a more relevant player. A motif associated with the transcriptional repressor zinc finger protein 148 (ZNF148), was significantly enriched in downregulated promoters (S3C Fig), suggesting that ZNF148’s repressor activity increased post-infection. The repressor E2F5’s motif was also significantly enriched in downregulated promoters (p-value = 0.0038). It has been shown that E2F5 is inhibited by retinoblastoma protein 1, which is directly inhibited by the KSHV protein, LANA [51]. These data support that motif enrichment scores can successfully denote prediction of transcription factor activity for use in the analyses below.

### Integration of proteomic and transcriptomic data of endothelial cells latently infected with KSHV into a single network model

To build a comprehensive network model that describes the host response to viral infection, we used the Prize-Collecting Steiner forest algorithm to integrate the proteomic and TF motif analyses [52]. This algorithm parsimoniously identifies the protein-protein interaction most likely to be relevant for connecting the relevant factors identified in the two types of analyses. In addition, it identifies Steiner nodes, which are proteins that were not implicated in the proteomic or TF analyses but form crucial connections between other important proteins identified as altered by KSHV in the global data sets generated. The proteomic data was integrated with the TF enrichment scores rather than the differentially expressed genes from the RNAseq data because TF transcript levels do not necessarily reflect regulatory activity [53]. When combining gene expression data with other types of protein scores for pathway reconstruction, it is therefore preferable to use inferred TF activities [28, 29, 32, 54]. The complete predicted Steiner forest network is large, connecting hundreds of proteins that respond to KSHV infection and the TFs inferred to regulate the transcriptional changes (S4 Fig). Randomization analysis shows that the selected proteins and interactions are specific to KSHV infection and do not reflect biases in the protein-protein interaction network or Steiner forest algorithm (S7A and S7B Fig).

**Fig 3:**
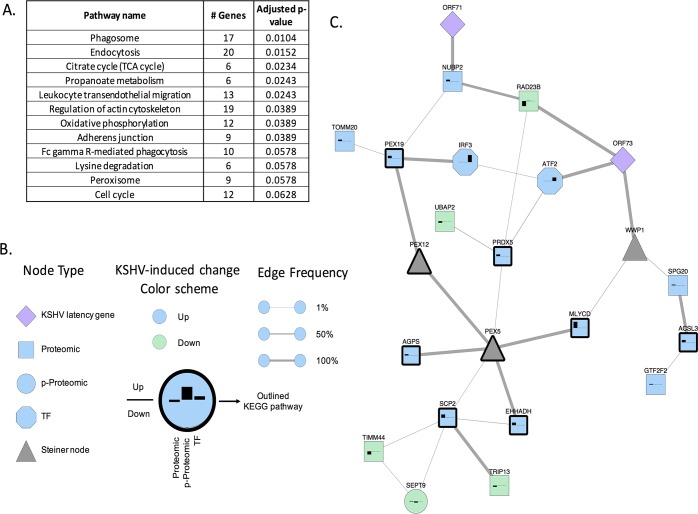
Steiner forest prediction of pathways activated by KSHV infection. **(A.)** List of the top KEGG pathways predicted to be altered by KSHV infection of endothelial cells from Steiner forest network and KEGG pathways database analysis (complete list in S1 Table). **(B.)** The bar graph on each node displays a signed version of the prizes used as input for the Steiner forest analysis. Each bar from left to right indicates the proteomic, phosphoproteomic (p-Proteomic), and TF scores. Positive scores (above the horizontal line) indicate the protein had higher intensity or the TF’s target genes were more highly expressed in KSHV infection than mock infection. Negative scores indicate that KSHV infection decreases intensity or lowers activity. Node color indicates whether the protein primarily upregulated (blue) or downregulated in response to viral infection (green). Node shape indicates the largest score for that protein: proteomic (square), phosphoproteomic (circle), TF (octagon), or Steiner node with no score (triangle). Latency-related KSHV genes are shown as purple diamonds. Steiner nodes are shown in gray. Edge thickness indicates the fraction of Steiner forest networks that contain the edge when the algorithm is run multiple times. **(C.)** Proteins with bold borders are peroxisome pathway members: SCP2, PRDX5, ACSL3, MLYCD, AGPS, EHHADH, PEX19 and two predicted Steiner nodes, PEX12 and PEX5. Their direct neighbors in our KSHV network are displayed as well to show their relationships. A Full Steiner forest is shown in figure S4 Fig.

To focus on specific biological functions, we assessed the overlap between the proteins in our Steiner forest network and KEGG pathways. We required a minimum overlap of 5 proteins and used the Benjamini and Hochberg multiple hypothesis test correction (FDR < 10%). The significantly enriched pathways included pathways involved in phagocytosis, immune response and several metabolic processes among others (Fig 3A and S1 Table for complete list). Our lab has shown that metabolism is altered during latent KSHV infection, including carbon and lipid metabolism, which supports our integrated network analysis [19, 22, 48]. There are several interesting pathways that are predicted to be altered during KSHV latent infection (S1 Table). From this analysis, we decided to follow up on proteins that clustered together, particularly those involving peroxisome metabolic lipid signaling. We have previously identified that lipid metabolism is required during latency [19], but how these metabolites are further utilized during KSHV latent infection still unknown. Therefore, we chose to further analyze activation of peroxisomes by KSHV. Peroxisome related proteins identified in the subnetwork including SCP2, PRDX5, ACSL3, MLYCD, AGPS, EHHADH, PEX19 were upregulated following KSHV infection and two Steiner nodes PEX12 and PEX5 were predicted by the algorithm to be activated by KSHV (Fig 3C). The proteins in this cluster are involved in lipid metabolism (SCP2, ACSL3, MLYCD, AGPS, EHHADH) and peroxisome organelle biogenesis and transport (PEX19, PEX12 and PEX5). Therefore, this sub- network predicts that KSHV induces peroxisome pathways involved in lipid metabolism and biogenesis. In addition, IRF3 is an interferon inducible gene activator that was predicted in the TF analysis to have increased transcriptional activity. It is not annotated as a peroxisome pathway protein, but the Steiner forest algorithm includes IRF3 in our peroxisome subnetwork due to its predicted relationship with PEX19 and previous evidence of a direct IRF3-PEX19 interaction [55] cataloged in the iRefIndex database. This observation suggests that peroxisomes might also play a role in immune signaling during latency, which might be an important regulatory control point of KSHV infection.

In addition, we incorporated a protein-protein interaction database from a study that mapped global interactions between KSHV genes and host proteins using viral gene pulldowns [56]. Since our study is mainly focused on latency, we included only the KSHV latent viral proteins and host proteins hits with our predictive Steiner forest network. From the database, we found that two viral genes have been shown to interact with proteins associated with peroxisome biogenesis. The latent KSHV proteins that are predicted to be associated with peroxisome biogenesis are shown as purple diamonds in Fig 3C. The KSHV protein-protein interaction database used total protein pull downs and therefore does not demonstrate direct interactions, rather it shows associations with the identified protein. All the proteins from the KSHV major latent locus were included in the Steiner forest analysis shown in figure S5 Fig. The utilization of KSHV protein-protein interaction dataset with the other protein-protein interaction databases advances our predictions of pathways that could be important to KSHV pathogenesis.

### KSHV upregulates peroxisome formation during latent infection

**Fig 4:**
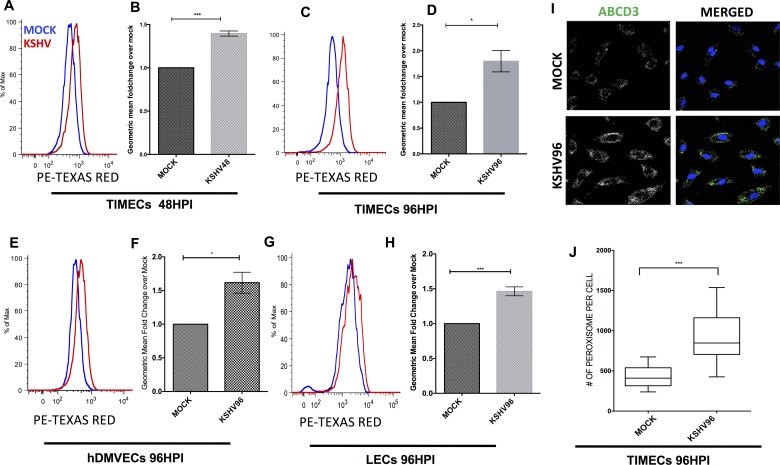
KSHV latently infected endothelial cells induces peroxisome formation. **(A.)** Flow cytometry of mock- and KSHV- infected TIME cells (TIMECs) harvested at 48 hpi, fixed and stained with antibody to ABCD3, a peroxisome marker. **(B.)** Geometric mean fold change of KSHV over mock at 48hpi for 3 experiments as in panel A, p < 0.05 student’s t-test. **(C.) Flow cytometry of** mock- and KSHV- infected TIMECs, harvested at 96 hpi, fixed and stained as in panel A. **(D.)** Geometric mean Fold change of KSHV over mock at 96 hpi for 3 experiments as in panel C, p < .05 student’s t-test. **(E.)** Flow cytometry of mock- and KSHV- infected primary human dermal microvascular endothelial cells (hDMVECs) harvested at 96 hpi, fixed and stained as in A. **(F.)** Geometric mean fold change of KSHV over mock at 96 hpi for 3 experiments as in E, p < .05 student’s t-test. **(G.)** Flow cytometry of mock- and KSHV- infected lymphatic endothelial cells (LECs) harvested at 96 hpi, fixed and stained as in A. **(H.)** Geometric mean fold change of KSHV over mock at 96 hpi for 3 experiments as in panel B, p < .05 student’s t-test. All the data are represented as mean +/- SEM and were analyzed using FlowJo software. **(I)** Representative confocal images of Mock and KSHV infected TIME cells at 96 hpi stained with antibody to ABCD3 and DAPI to identify the nuclei. **(J)** Quantification of number of peroxisomes per cell in three biological replicates of Mock and KSHV-infected cells stained as in panel I, analyzed using student’s t-test p < 0.0001.

To validate the prediction that peroxisome pathways involved in lipid metabolism and likely peroxisome biogenesis are increased during latent infection, we examined the protein levels of ABCD3, an ATP Binding Cassette Subfamily D Member 3, a lipid transporter specific to peroxisomes and a common marker to study peroxisomes, using flow cytometry at 48 and 96 hpi (Fig 4A–4H). Staining with an antibody to ABCD3 showed a significant increase in fluorescent staining in the KSHV infected cells compared to mock infected cells in three different infections at 48 and 96 hpi in TIME cells and 96 hpi in primary human dermal microvascular endothelial cells (hDMVECs) and lymphatic endothelial cells (LECs) (Fig 4E–4H), indicating that during latent infection KSHV significantly upregulates ABCD3 protein expression. In addition, we evaluated MLYCD and PEX19 and a non-clustered peroxisome protein PEX3 levels, in TIMECs, hDMVECs and LECs. PEX3 is a PEX19 docking factor required for PEX19 to deliver proteins into the peroxisome matrix [57]. Staining with MLYCD and PEX3 antibody showed a significant increase of the protein levels in KSHV infected TIME cells, primary hDMVECs and primary LECs compared to mock infected at 96 hpi (S6 Fig) while PEX19 was significantly upregulated in TIME cells (S6 Fig).

We next evaluated the peroxisome organelle number using confocal imaging analysis of mock and KSHV infected TIME cells stained with antibody to ABCD3. Peroxisome size ranges approximately between .4–1 uM. We evaluated 3D particles using z-stacks imaging and then quantified the particle number as a proxy of peroxisome organelle per cell with a minimum threshold of .5 uM. There is an approximately 50% increase in the number of peroxisomes per cell in the KSHV infected endothelial cells as compared to mock infected cells (Fig 4I and 4J). Representative pictures of the mock and KSHV infected cells stained with an antibody to ABCD3 are shown in Fig 4I. Combined, these observations support the prediction from the Steiner forest analysis that KSHV promotes peroxisome biogenesis.

### KSHV latent locus is sufficient to induce peroxisome lipid transporter

**Fig 5:**
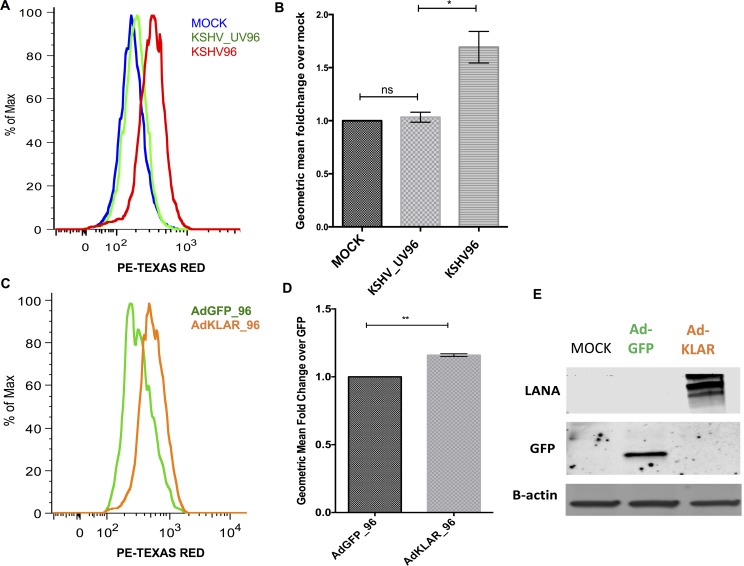
KSHV latency locus is sufficient to induce a peroxisome marker in endothelial cells. **(A.)** Flow cytometry of mock-, KSHV-UV irradiated and KSHV- infected TIME cells harvested at 96 hpi, fixed and stained with antibody to ABCD3. **(B.)** Geometric mean fold change of KSHV, KSHV-UV-irradiated over mock at 96 hpi for 3 experiments as in A, p < .05 student’s t -test. **(C.)** Flow cytometry of AdGFP or AdKLAR (KSHV latency-associated region in a gutted adenovirus) infected TIME cells harvested at 96 hpi, fixed and stained with antibody to ABCD3. **(D.)** Geometric mean fold change of AdGFP over AdKLAR at 96 hpi p < .05 student’s t-test. All the data are represented as mean +/- SEM and were analyzed using FlowJo software. (E.) Western blot analysis of TIME cells mock infected or infected with AdGFP-, or AdKLAR stained with antibodies to GFP or LANA.

To determine that the increase of peroxisome numbers per cell was not a cellular response to infection but rather induced by virally encoded genes, cells infected with UV irradiated KSHV were stained with ABCD3 antibody and measured by flow cytometry. UV irradiated virus can bind and enter cells but does not express viral genes. Flow cytometry analysis showed no increase in the expression of ABCD3 following infection with UV irradiated virus (Fig 5A and 5B). Therefore, the increase of ABCD3 in latently infected cells requires KSHV gene expression and it is not a cellular response to virus entry into the cell.

The KSHV latent locus is comprised of LANA, vCyclin, vFlip, Kaposins and 12 microRNA loci. To assess the role of the KSHV latent locus in increasing the number of peroxisomes, we evaluated whether the KSHV latency associated region (KLAR) is sufficient to induce the increase of ABCD3 protein expression levels. The KLAR locus (a kind gift from Dr. Rolf Renne) was cloned into a helper-dependent gutted adenovirus vector that does not express any adenovirus genes. Cells were infected with a control gutted adenovirus (Ad) only expressing GFP (AdGFP) and the gutted adenovirus expressing KLAR (AdKLAR) and stained with ABCD3 antibody. Infection rates for AdGFP and AdKLAR were 59% and 97% respectively as determined by expression of GFP or LANA. To adjust for the differences in the infection rates, we gated on the GFP positive cells from the AdGFP infected cells and then compared to the AdKLAR cells. Cells infected with the AdKLAR expressing gutted adenovirus exhibited increased ABCD3 protein expression compared to mock infected cells and AdGFP (Fig 5C and 5D). Therefore, the latency genes are sufficient to induce ABCD3 protein expression levels.

### KSHV requires peroxisome proteins involved in lipid metabolism during latent infection

**Fig 6:**
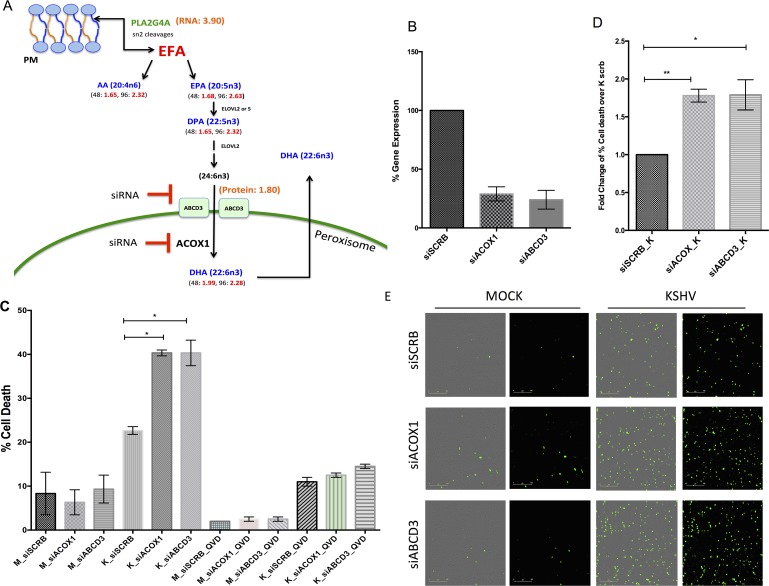
KSHV latently infected endothelial cells require peroxisome proteins. **(A.)** Overview of Essential Fatty Acids (EFA) and peroxisome pathway. Numbers indicate genes altered by KSHV (time post infection in black and fold change in red) as identified by RNA-seq in orange (*PLA2G4A*), a previously published metabolomics screen [19] in blue and proteomic screen in red. siRNA treatments of ABCD3 and ACOX1 for panel B are indicated in blocked red sign. (B.) TIME cells were transfected with a control siRNA (siSCRB) or siRNA to ABCD3 or ACOX1. siABCD3 and siACOX1 treatments lead to greater than 70% reduction in ABCD3 and ACOX1 expression as determined by qRT-PCR normalized to the housekeeping genes GAPDH and HPRT. **(C.)** TIME cells were transfected with siRNAs as in panel B and 24 later were Mock- or KSHV-infected. 96 hpi (120 hours post transfection) cells were harvested and % cell death was measured using Trypan blue stain. In parallel, cells were treated with 20 μM QVD, a pan-caspase inhibitor. Data shown is from three independent experiments. Student’s t-test (**D**.) Data shows the average fold change in % dead cells over control siRNA transfected cells from three independent experiments from panel C. **(E.)** IncuCyte microscopy images identifying dead cell nuclei (YOYO-1) for Mock- and KSHV-infected cells transfected with siSCRB, siABCD3 or siACOX1 at 96 hpi. Essen software was used to identify cell nuclei by size and fluorescent intensity, with background subtracted. YOYO-1 positive nuclei are in fluorescent green. All the data are represented as mean +/- SEM.

Peroxisomes are involved in lipid signaling and metabolism. Our previous metabolomics screen indicated that several lipid metabolites are altered by KSHV during latent infection of endothelial cells, including two metabolites generated in the peroxisome, dihydroxyacetone phosphate (DHA-P) and docosahexaenoate (DHA; 22:6n3) [19] and metabolites upstream of DHA are also upregulated as indicated in red numbers (Fig 6A). DHA is synthesized from 24:6n3 by Acyl-CoA Oxidase 1 (ACOX1) an enzyme mainly expressed in the peroxisome and it is involved in the first step of peroxisomal β-oxidation [43] (Fig 6A). To determine if ACOX1 is necessary during KSHV latent infection, small interfering RNA (siRNA) was used to knockdown its gene expression (Fig 6B). Loss of ACOX1 did not alter the cellular proliferation of uninfected cells or the KSHV infection rates but resulted in a significant increase in cell death of the KSHV infected cells compared to controls at 96 hpi (Fig 6C–6E). As ACOX1 is the main enzyme involved in metabolizing DHA, these observations suggest that DHA might be required during infection.

The precursor of DHA, 24:6n3 is transported into the peroxisome by the lipid transporter ABCD3 [34, 58]. Therefore, we evaluated if ABCD3 is required during latency by transiently silencing its gene expression. Similarly, to ACOX1, loss of ABCD3 did not alter cellular proliferation of uninfected cells or KSHV infection rates but resulted in a significant increase in cell death of the KSHV infected cells compared to controls at 96 hpi (Fig 6C–6E). Therefore, both ACOX1 and the ABCD3 transporter are required for the survival of endothelial cells latently infected with KSHV. In parallel, we treated cells with a pan-caspase inhibitor, QVD, to test whether apoptosis was the main cell death mechanism. KSHV-siABCD3 and KSHV-siACOX1 cells treated with QVD showed a 3-fold decrease in cell death, indicating that apoptosis was the main cell death mechanism (Fig 6D). Therefore, this data strongly indicates that peroxisomal proteins involved in lipid metabolism are required for the survival of endothelial cells latently infected with KSHV.

## Discussion

**Fig 7:**
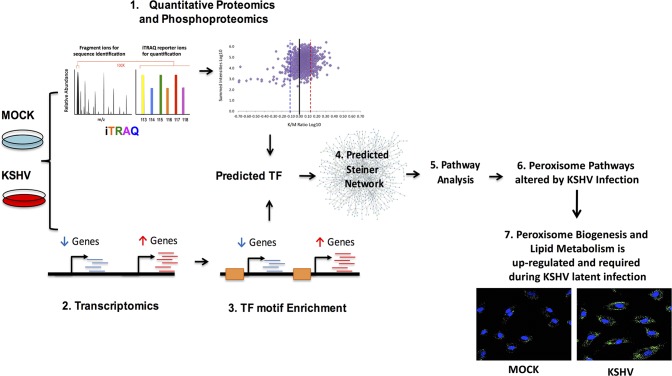
Workflow of the systems biology data integration analysis and Schematic of the metabolism of VLCFs in the peroxisome. Experimental conditions of mock and KSHV infected TIME cells were processed using proteomics techniques and in parallel transcriptomics analysis. The RNA-seq data was used for TF prediction and then TFs were used as the link to generate the predicted protein-protein interaction Steiner forest network. Analysis of the Steiner forest network was done using KEGG pathway analysis and followed by experimental validation of peroxisome biogenesis and mediated lipid metabolism.

We integrated transcriptomics, proteomics and metabolomics analyses, to provide a comprehensive view of cell signaling in an oncogenic virus infection in human endothelial cells, the cell type likely to be most relevant to KS tumor cells (Fig 7). From quantitative measurements of the phosphoproteome and proteome analysis of endothelial cells latently infected with KSHV, we found that latent infection alters the levels of at least 289 proteins, approximately 13% of the proteome quantified, as well as 192 altered phosphorylation sites, approximately 12% of the phosphosites quantified in this study. Previous studies using mass spectrometry based proteomics and KSHV, was done with targeted proteomics to identify protein-protein interactions specific to single viral proteins, LANA and K5 for example, using immunoprecipitation and 2D-gel mass spectrometry [59–69]. Our dataset is the first that we are aware of, that analyzes the global response to latent KSHV infection with both phosphoprotein and proteomic studies in endothelial cells. The list of phosphosites altered by KSHV infection may provide deeper insights into cell signaling activation following KSHV infection of endothelial cells and should serve as a useful dataset for future studies. From transcriptomics analysis, we found that KSHV infection leads to alterations in approximately 17% of the host cellular transcriptome. This dataset was generated using next generation sequencing providing more comprehensive gene expression profiles in endothelial cells latently infected with KSHV than previously published. Transcriptomic analysis of KSHV infection has been done in KS tumors and in PEL cells, but only older microarray technology for endothelial cells has been previously done [70–78]. The activity of several TFs was predicted to be activated or repressed by latent infection as identified from transcription factor motifs found in the promoters of host genes that were up or down regulated following KSHV infection of endothelial cells. These TFs serve as a link to map protein-protein interactions, connecting upstream signaling to downstream gene-expression targets. The goal of this integrated systems biology approach was to identify novel pathways that could not be predicted by one platform alone. Various functional networks, including phagosomes, endocytosis and multiple metabolic pathways including peroxisome biogenesis were identified by the Steiner forest analysis providing a rich data set for future studies. We chose to further analyze peroxisome biogenesis as peroxisomes are involved in several pathways likely to be important for KSHV pathogenesis including redox control and the breakdown of very long chain fatty acids. A sub-network cluster of peroxisomal proteins predicted to be activated by the Steiner forest analysis is shown in Fig 3C. The presence of this sub- network implies increased peroxisome activity in KSHV latently infected endothelial cells. The integrated analysis is further substantiated by a significant increase in the number of peroxisomes per cell during KSHV latency, induced specifically by KSHV latent gene expression as opposed to a cellular response to a viral infection. Upregulation of peroxisomes was further validated by identifying the upregulation of several peroxisomal proteins in TIME cells, primary dermal microvascular endothelial cells as well as in primary lymphatic dermal microvascular endothelial cells, the cell type that most closely resembles KS spindle cells [78].

We previously found that KSHV latent infection dramatically alters the lipid profile of endothelial cells [19]. In the KSHV infected cells, there was a significant increase in most of the LCFAs measured. We also found that FAs synthesis was necessary for the survival of endothelial cells latently infected with KSHV [19]. In our metabolomic screen we also noted that DHA and its precursors, as well as DHA-P, were increased following KSHV infection during latency [19] (Fig 6A). DHA is an important metabolite involved in anti-inflammatory responses and cellular development and is mainly produced in the peroxisome by partial β-oxidation [43, 44, 79]. Knockdown of ACOX1, the enzyme that produces DHA, results in a significant increase in the death rate of latently infected endothelial cells but not their mock infected counterparts. Furthermore, the peroxisome-specific lipid transporter ABCD3, which transports VLCFAs including a precursor of DHA, is also essential for KSHV-infected endothelial cell survival. Therefore, lipid metabolism in the peroxisome is essential for the survival of endothelial cells latently infected with KSHV.

Peroxisomes appear to play a role in the response to lytic viral infection for several viruses. This organelle serves as a signaling platform for antiviral response against unrelated non-enveloped and lipid-enveloped RNA and DNA viruses including Reovirus, Sendai virus, Dengue virus and Influenza virus in infected mouse embryonic fibroblast cells [36–39]. Interestingly, one study established that Influenza virus modulates and requires peroxisomal ether lipid metabolism for efficient virion replication in A549 epithelial cells [37]. These observations underscore complex and sometimes paradoxical cellular changes that involve peroxisomes during viral infection; while Influenza requires peroxisome metabolism for virion production, peroxisomes also play an important role in the immune response triggered by infection. The interplay between peroxisome responses and viral infection may depend on the cellular environment and virus type. Our study elucidates a novel mechanism by which a latent herpesvirus infection manipulates peroxisomal lipid signaling required for survival in a long-term infection.

KSHV is known to activate COX-2/PGE2/EP vector during *de novo* infection mediating an underlying pro-inflammatory state conducive to long-term latency [21]. COX-2 converts Arachidonic Acid (20:4n6, or AA) to PGE2, which then regulates autocrine and paracrine signaling. Our previously published metabolomics screen demonstrates that KSHV latent infection upregulates precursors of the COX-2/PGE2/EP signaling, such as AA indicating that signaling upstream of the COX-2/PGE2/EP pathway is active during latency. Furthermore, upregulation of DHA-P and DHA as shown by our metabolomics screen indicates, that the peroxisomes are enzymatically active and producing these metabolites [33]. Therefore, the peroxisome represents a crossroads of lipid signaling and bridges the gap between upstream essential fatty acids (AA, EPA and DPA) and how they are metabolized downstream (DHAP, DHA) during latent KSHV infection (Fig 6A).

AA is a pro-inflammatory metabolite and DHA has been associated with anti- inflammatory responses [80]; however, both are upregulated during latency. The KS tumor environment is characterized by a chronic inflammatory state [11]. Therefore, we hypothesize that KSHV commandeers control of cellular metabolic pathways to fine-tune a higher level of chronic inflammation by altering homeostatic mechanisms and maintaining a shifted equilibrium in this new inflammatory state required for the maintenance of latency. Further work is required to elucidate whether the primary role of peroxisomes is to regulate lipid signaling and inflammation, if peroxisomal ether lipid metabolism is required or if peroxisomes are also involved in regulating H2O2, which often occurs in parallel. It has been shown that in primary endothelial cells during exogenous stress, inflammatory cytokine expression is downregulated by using DHA as anti-inflammatory treatment [81]. It would be interesting to determine if altering DHA synthesis influences inflammatory signaling proteins in endothelial cells latently infected with KSHV.

Currently, pharmacological approaches that target herpesvirus infection focus on lytic replication and there are no treatments specific for latent infections. Since KS tumors primarily exhibit latent infection, this study elucidates critical control point mechanisms in the latent phase offering an understanding of KSHV viral pathogenesis and provides potential novel and combinatorial molecular therapeutic targets through large scale identification of pathways activated by KSHV latent infection of endothelial cells.

## Materials and methods

### Reagents, cell, viruses and infection

QVD-OPH (SMBiochemicals) was dissolved in DMSO and used at a final concentration of 20 μM. YOYO-1 and SytoGreen were purchased from Thermofisher scientific. Tert-immortalized microvascular endothelial (TIME) cells were obtained from the McMahon lab and previously described in Venetsanakos, et. al. [82], human dermal microvascular endothelial cells (hDMVECs) and lymphatic endothelial cells (LECs) (LONZA Walkersville, MD) were maintained as monolayer cultures in EBM-2 media (LONZA Walkersville, MD) supplemented with a bullet kit containing 5% FBS, vascular endothelial growth factor, basic fibroblast growth factor, insulin-like growth factor 3, epidermal growth, and hydrocortisone. KSHV for phosphoproteomic, proteomic and transcriptomic experiments was purified from BCBL-1 cells, a primary effusion lymphoma cell line that maintains wild type KSHV, as described previously [22]. For most of the subsequent studies, KSHV was isolated from iSLK cells containing a recombinant KSHV made from KSHV-Bac16 containing the GFP gene as described previously [83]. For all experiments KSHV was titered and used to infect TIME cells as previously described [46]. Infections were performed in serum-free EBM-2 media and subsequently overlaid with complete EBM-2 media. Infection rates were assessed for each experiment by immunofluorescence and only experiments where greater than 90% of the cells expressed LANA, a latent marker, and less than 2% of the cells expressed ORF59, a lytic marker, were used as previously described [46].

### Helper dependent adenovirus expressing GFP and KLAR

To express the KSHV latent genes in the absence of other viral gene expression, The 12.6 kbp KSHV latency associated region (KLAR) containing the native LANA promoter, LANA, vCyc, vFLIP, all 12 miRNA loci and the kaposins through the native polyadenylation signal downstream of the kaposins, was obtained from the Renne lab. The helper dependent Adenovirus contains the adenovirus packaging signal but no adenovirus genes and was purchased from MicroBix. To create AdKLAR and AdGFP, the KSHV KLAR region or GFP was cloned into a shuttle vector (pBShuttle) flanked by adenoviral sequences. The KLAR/adenovirus expression cassette was then excised from this plasmid and electroporated into BJ5183 cells (Stratagene) along with pC4Hsu helper adenovirus vector (Microbix Biosystems) to allow for homologous recombination. The resulting plasmid (AdKLAR or AdGFP) was transfected into 293Cre cells, which stably express a Cre recombinase enzyme, selectable with puromycin. Cells were passaged in the presence of helper adenovirus (HD14; Microbix), which contains the adenovirus coding regions and allows to produce AdKLAR adenovirus. The Helper Adenovirus contains a modified packaging sequence flanked by loxP sites; therefore, the helper adenovirus is not packaged due to an excision of the packaging sequences. After expansion of the adenovirus, cells were collected, pelleted, and freeze-thawed three times using liquid nitrogen and 37 C water bath. Cell debris was spun out at 2000rpm and the cell-free supernatant was collected. The cleared lysate was layered onto a continuous 15% to 40% CsCl gradient and centrifuged for 2–3 hours at 35,000g using a SW41Ti rotor (Beckman Coulter, Inc., Fullerton, CA). The mature virus band was collected and purified in a second CsCl density gradient. The virus band was collected, dialyzed against three changes of A195 buffer. Infections were performed in serum-free EBM-2 medium supplemented with 1μg/mL poly-L-lysine for 1 hour, after which the medium was replaced with complete EGM-2 media. Infection rates were assessed for each experiment by immunofluorescence for LANA, a latent marker, and GFP for AdGFP expression.

### Flow cytometry cell analysis

Mock-, KSHV-infected cells were washed with PBS and removed by trypsinization, fixed, with 4% paraformaldehyde for 30 min on ice and processed for flow cytometry. Cells were permeabilized and blocked with .1% triton and 1% NGS. The ABCD3 transporter was detected with the PMP70 (ABCD3) antibody from thermoscientific product# PA 1–650, MLYCD Proteintech Group (15265-1-AP), PEX3 Novus a Biotechne brand (NBP1-86210) and PEX19 Abcam (ab 137072). After staining with the primary antibody for 1 hour, the cells were reacted with a secondary Alexa Fluor 594 and Alexa Fluor 488 both anti-rabbit (ThermoFischer P#A11072) antibodies. Samples were analyzed by FLOWJO, flow cytometry analysis software.

### Confocal microscopy

TIME cells were seeded on a 4-well glass chamber and were processed for confocal microscopy by fixing in paraformaldehyde (4% in 1XPBS) at 37°C. Samples were permeabilized with Triton X-100 (0.5% in 1XPBS). Incubations with primary antibodies diluted (1:1,000) in blocking buffer (3% bovine serum albumin [BSA] and 1XPBS) were carried out at room temperature (RT) for 30 minutes. Samples were then incubated with secondary antibodies (Alexa Fluor 488 anti-rabbit) in blocking buffer for 25 min at RT. Prior to mounting; samples were incubated with DAPI for 5 min at RT coverslips were mounted on microscope slides. Confocal images were acquired using Zeiss LSM 510 Meta confocal microscope Olympus.

### Quantification of peroxisome numbers

2-5um Z-stacks were acquired using a Zeiss 510 META confocal microscope equipped with a 63X / 1.4 NA Oil DIC objective. The exported images were then processed using Imaris 7.2.3 software (Bitplane) for peroxisome quantification and ImageJ was use for figure images. Cytoplasmic peroxisomes were quantified based on voxels graphics. The data were then analyzed using student’s t-tests.

### siRNA transfection and cell death assay

siRNAs specifically targeting ACOX1 (pre-validated) were purchased from Santa Cruz Biotechnology (Cat. Sc-94104). A negative-control siRNA (siSCRB) and ABCD3 (pre-validated) were designed and synthesized by Ambion. TIME cells were transfected with siRNA using the Amaxa Nucleofector Kit by Lonza per the manufacturer’s protocol. At 24 hour post transfection, cells were Mock- or KSHV-infected. At 96 hpi cell death was measured using Trypan blue assay and cell were quantified using TC20 cell counter from BioRad. In parallel, cell death fluorescent images were acquired using the IncuCyte from Essen Bioscience using YOYO-1 or SytoGreen (both probes from Thermofisher scientific).

### Sample preparation for phosphoproteomic analysis

Approximately 5 million cells were lysed in 2 mL of 8M Urea. Protein concentration was determined by the BCA assay (Pierce). Samples were reduced with 5 mM dithiothreitol at 56 C for 1 hour, and then alkylated with 15 mM iodoacetamide for 1 hour at RT in the dark. Samples were diluted 4-fold with 100 mM Ammonium Acetate, pH 8.9, and digested with Sequencing Grade Modified Trypsin (Promega) at a ratio of 1:100 (trypsin to total protein), overnight at RT. Following digestion, peptides were desalted and concentrated using Sep-Pak Plus C18 cartridges (Waters, cat. no. WAT020515) per the manufacturer’s recommendations. Samples were then dried by vacuum centrifugation, lyophilized, and stored at -80 C until further processing.

### Isobaric labeling of peptide samples

Phosphorylated samples were labeled with 8-plex iTRAQ reagents (AB Sciex). Lyophilized peptides derived from approximately 1 million cells were resuspended in 30 uL of dissolution buffer (0.5 M N(Et)3HCO3 pH 8.5–9). iTRAQ labels were resuspended in 70 uL of isopropanol and added to the peptide mixture. Samples were incubated at RT for 2 hours, combined, and dried overnight by vacuum centrifugation. The following day, samples were desalted and concentrated using Sep-Pak Vac 1cc (50mg) cartridges (Waters, cat. no. WAT054955) according to the manufacturer’s recommendations. Samples were then dried by vacuum centrifugation, lyophilized, and stored at -80 C until further processing.

### IMAC and phosphotyrosine enrichment

Approximately 100 uL of packed Ni beads (Ni-NTA Superflow beads, Qiagen) were washed three times in water and stripped with 100 mM EDTA pH ~8.9 for 30 min. Beads were then washed three times with water and once with 80% ACN in 0.1% trifluoroacetic acid. Lyophilized iTRAQ samples were resuspended in 1.5 mL of ACN in 0.2% TFA and incubated with prepared beads for 1 hour at RT. Beads were then washed three times with 80% ACN in 0.1% TFA, and phosphopeptides were eluted from beads with 2 incubations in 75 uL of 1.4% Ammonia. Samples were then vacuum centrifuged down to ~20 uL. 2 uL of 200 mM ammonium formate pH 10 was added and samples were directly analyzed by mass spectrometry.

### Mass spectrometry

Peptide samples were loaded onto a first-dimension trap column (Waters Xbridge, C18, 10 uM particle size, 100 Å pore size, 4 cm packing length 150 uM column inner diameter). Online peptide separation coupled to MS/MS was performed with a 2D-nanoLC system (nanoAcquity UPLC system, Waters) and a Velos-Pro/Orbitrap-Elite hybrid mass spectrometer (ThermoFisher Scientific). Six discrete elutions were performed at 1.5 uL/min with 5mM ammonium formate pH 10 using increasing concentrations of ACN (1%, 3%, 6%, 15%, 25% and 44%) and diluted with 6 uL/min 0.1% formic acid (FA) prior to loading onto a second dimension trap column (Dr. Maisch ReproSil-Pur, C18, 5 uM particle size, 120 Å pore size, 4 cm packing length 150 uM column inner diameter) connected to an analytical column (Orochem Reliasil, C18, 3 uM particle size, 90 Å pore size, 20–25 cm packing length 50 uM column inner diameter) with an incorporated electrospray emitter. Peptide separation was achieved using a gradient from 3 to 80% (V/V) of ACN in 0.1% FA over 115 minutes at a flow rate of 200 nL/min. The mass spectrometer was operated in data-dependent mode using a Top 10 method. Full MS scans (m/z 300–2000) were acquired in the Orbitrap analyzer (resolution = 120,000), followed by high energy collision induced dissociation (HCD) MS/MS (fm/z 100–2000, resolution = 15,000) at a normalized collision energy of 35%.

### Data processing

MS data files were searched using the COMET [24, 84] algorithm and the output was imported into the Trans-Proteomic Pipeline [85] with the following parameters: variable oxidation of methionine, variable phosphorylation of Serine, Threonine, or Tyrosine, up to 4 variable modifications per peptide, fixed oxidation of Cysteine, and fixed iTRAQ labeling of Lysines and the N-terminus, maximum charge of 7. Peptide false discovery rate (FDR) was set to 5% for phosphorylation analysis. Peptide quantification based on the iTRAQ labels was determined using the LIBRA software embedded in the Trans-Proteomic Pipeline. Phosphopeptides were normalized to an internal control peptide (VNQIGpTLSESIK) from the enolase digest containing phosphorylated peptides. For each biological replicate, 2 technical replicates were run. A total of 12 fractions for the phosphoproteome, 12 fractions for the proteome and 2 fractions for phosphotyrosine- enriched runs were analyzed. 3,579 peptide spectra profiles were analyzed for the proteome, 4982 for the phosphoproteome including the phosphotyrosine residues. From the phosphotyrosine enrichment 1053 total spectra were analyzed. There was approximately 70% overlap of proteins identified from both technical runs and approximately 50% overlap in the phosphoproteome (S2A Fig).

Each peptide included in the analysis was identified in a minimum of 2 spectra, and each protein included in the analysis was identified by a minimum of two unique peptides. To deconvolve complex overlapping spectra profiles, we used the Hardklör algorithm [86, 87] in conjunction with the MassSpecUtil tool to merge spectra for iTRAQ analysis quantification. This was done to separate any possible overlapping isotopic envelope and providing better peptide identification. Comet search algorithm was used to identify the peptide spectra with a 5% FDR.

To assess significantly changing protein phosphorylation and abundance, peptide-spectrum matches that did not have an intensity of at least 10 in all channels were removed. All channels were median normalized, and the intensities were summed over all peptides of the same protein for each condition. The KSHV-specific effects were assessed by computing the log2(K/M) fold change for the means of the KSHV- (K) and mock (M)-infected biological replicates. A paired t- test was used to calculate the significance of the changes. Each technical replicate was analyzed independently.

### RNA-sequencing

TIME cells were Mock- or KSHV-infected with virus isolated from BCBL-1 cells, as described above, and incubated for 48 hours. Total mRNA was isolated from TIME cells using the NucleoSpin RNA kit (Machery-Nagle, Bethlehem, PA). mRNA was further concentrated and purified using the RNA Clean and Concentrator kit (Zymo Research, Irvine, CA). Purified mRNA samples were processed at the Benaroya Research Institute Genomics core facility and sequenced using an Illumina HiSeq 2500. Image analysis and base calling were performed using RTA v1.17 software (Illumina). Reads were aligned to the Ensembl's GRCh37 release 70 reference genome using TopHat v2.08b and Bowtie 1.0.0 [88, 89]. Counts for each gene were generated using htseq-count v0.5.3p9. The data have been deposited in NCBI's Gene Expression Omnibus [90] and are accessible through GEO Series accession number GSE84237.

### Motif scanning using FIMO

DNA sequences 1000bp upstream of annotated transcription start sites were downloaded from the Genome Reference Consortium at http://hgdownload.cse.ucsc.edu/goldenpath/hg19/bigZips/upstream1000.fa.gz on May 29, 2015. Position-frequency matrices of 426 motifs were taken from a database of consensus motifs compiled from a variety of experimental techniques downloaded from http://meme-suite.org/meme-software/Databases/motifs/motif_databases.12.11.tgz. For each motif, FIMO software was used to find the top 1000 instances by p-value of each motif in the sequences. A motif was considered to flank a gene if an instance of the motif exists within 1000bp upstream of the gene’s transcription start site.

### Wilcoxon rank-sum tests for putative binding site enrichment

For each TF with at least 50 reads in all three mock-infected replicates or all three KSHV- infected replicates, a two-tailed Wilcoxon rank-sum test compared the change in expression post- infection of the genes flanked by of its motif locations to the change in expression of genes that are not. For the test, the genes were ranked by the significance and direction of their change in expression as analyzed by DESeq, where the highest-ranking genes were associated with low DESeq p-values and increased expression post-infection, the lowest-ranking genes were associated with low DESeq p-values and decreased expression, and the intermediate genes had high p-values. The Wilcoxon rank-sum tests compared the sum of the ranks of binding-site- flanked genes to a null normal model.

### Network analysis

We conducted network analysis with the Prize-Collecting Steiner Forest (PCSF) algorithm from the Omics Integrator package, which uses msgsteiner for optimization [91]. PCSF identifies a sparse sub-network that connects the proteins highlighted by mass spectrometry with the TFs identified by the motif-based analysis. It assigns positive scores (prizes) to the proteins and TFs that reflect how relevant they are to KSHV infection and edge costs to protein-protein interactions that represent how trustworthy they are, with reliable edges receiving lower costs. The sub-network maximizes the cumulative prizes of the included proteins while minimizing the edges costs. It can include Steiner nodes, which are proteins that were not assigned prizes but form critical links between other proteins. We created an endothelial-specific weighted interaction network by integrating our RNA-seq data with the iRefIndex PPI network [26].

The PCSF algorithm requires protein prizes that quantify how relevant they are to the biological process of interest and PPI edge costs that describe how reliable they are. To calculate protein prizes, we scaled the p-values from the proteomic and phosphoproteomic technical replicates into the range [0, 1] by computing -log10(p-value), subtracting the minimum value across all proteins, and dividing by the maximum value across all proteins. Each proteomic and phosphoproteomic replicate was scaled independently. For the TF prizes, we transformed the Wilcoxon rank-sum q-values as -log10(q-value) but did not rescale them. Visualizing the histograms of the proteomic and TF scores revealed that the proteomic scores would dominate the TF scores if the TF scores were rescaled. For each protein, we then selected the maximum score from the two proteomic technical replicates, two phosphoproteomic technical replicates, and TF motif scores, which produced prizes for 3080 unique proteins.

We used the iRefIndex (version 13.0) PPI network [26]. The iRefIndex database aggregates PPI from multiple primary interaction databases such as BioGRID [92], DIP [93], HPRD [94], and IntAct [95]. All edges represent direct, experimentally-detected physical interactions between two proteins as opposed to predicted PPI or other types of functional relationships. We calculated edge costs between 0 and 1 based on the interaction metadata such as the interaction type, experimental assay, and number of supporting publications as in Ceol *et al*. [96]. PPIs identified using reliable, low-throughput experiments (for example, co- crystallization) are assigned much lower costs than interactions detected in large-scale screens. Interactions reported in multiple publications similarly receive lower costs. Low cost edges are more likely to be selected by PCSF so the PCSF subnetwork preferentially includes trustworthy protein-protein relationships. We created an endothelial-specific network by removing all unexpressed genes from the iRefIndex PPI network, which initially contains interactions from many types of human cells and tissues. Originally, the network contained 175854 interactions among 15404 proteins. After filtering genes that were not expressed at 50 counts or greater in all six of the RNA-seq replicates, the endothelial-specific network contained 121059 interactions among 9489 genes. To select PCSF parameters, we performed a grid search testing all combinations of β from 0.25 to 5.0 with a step size of 0.25, μ from 0 to 1.0 with a step size of 0.005, and ω from 0.5 to 3.0 with a step size of 0.5. Under some parameter combinations, the hub node ubiquitin C (UBC) was directly connected to a large portion of genes in the network so we discarded all networks containing UBC. The parameters β = 4.75, μ = 0.02, and ω = 2.5 produced the largest network without UBC, and we used these parameters for further analysis. We ran PCSF 1000 times with these parameters and additionally set r = 0.01 to add random noise to the edge costs. The multiple executions of PCSF with random noise were used to identify parallel paths between proteins in the different runs. Our final network was the union of the 532 of these 1000 PCSF networks that did not contain UBC. The union network contained 1253 interactions among 734 proteins, of which 44 were Steiner nodes. For visualization, we calculated edge frequency as the fraction of the 532 PCSF networks that contain a particular edge. The edge thickness in the network figures reflects edge frequency in the collection of PCSF networks, not the original interaction confidence score in the PPI network.

To assess whether the PCSF subnetworks are specific to KSHV infection, we ran PCSF 1000 times with the same parameters and randomized protein prizes. Each random run reassigned the observed KSHV protein prizes to random proteins in the PPI network. We removed the random PCSF outputs that contained the hub node UBC and computed node and edge frequencies with the 131 remaining forests.

We supplemented the PCSF union network with KSHV-human protein-protein interactions obtained from VirHostNet 2.0 [56, 97] (downloaded January 7, 2016). We considered only latency-related KSHV genes as defined by Davis *et al*.: K1 (K1_HHV8P), K2 (VIL6_HHV8P), K12A (K12_HHV8P), K12B, K12C, ORF71 (VFLIP_HHV8P), ORF72 (VCYCL_HHV8P), and ORF73 (ORF73_HHV8P). We queried VirHostNet and the Davis *et al*. interactions for all host-virus PPI between these latency-related KSHV genes and any human protein in our PCSF network. We then added all the relevant KSHV-human interactions to our network figures and did not use the PCSF algorithm to filter these edges.

We used WebGestalt [98] to identify KEGG pathways [47] that are enriched for proteins in our PCSF network. Because the predicted network can only contain proteins from the initial iRefIndex interaction network, we used all proteins in the protein-protein interaction network as the reference set. We required a minimum overlap of 5 proteins and used the Benjamini and Hochberg multiple hypothesis test correction (FDR < 10%). To visualize the network regions that are relevant to the enriched KEGG pathways we used Cytoscape [99] to select all proteins in the enriched pathway and their directly connected neighbors in the PCSF network. We then included all KSHV- human PPI that involve the KEGG pathway members and their PCSF neighbors.

## Supporting information

S1 Fig Relative abundance plot of normalized mean intensities of iTRAQ labeling in the proteome and phosphoproteome. **(A.)** Phosphoproteome technical replicate (TR) 1. **(B.)** Phosphoproteome technical replicate (TR) 2. **(C.)** Proteome technical replicate (TR) 1. **(D.)** Proteome technical replicate (TR) 2.
(TIF)

S2 Fig Proteome and phosphoproteome profiling. **(A.)** Venn diagrams of both technical replicate runs from phosphoproteome and proteome. **(B.)** GO biological process analysis of the upregulated hits from the phosphoproteome and proteome measured proteins.
(TIFF)

S3 Fig **Transcriptomics profiling and motif enrichment comparisons (A.)** RNA-seq volcano plot. Highlighting the most highly upregulated and downregulated genes. **(B.)** Venn diagram of the genes inferred to have motif instances of IRF1, IRF2, STAT2, and IRF7 1000bp upstream of their transcription start site. The numbers show the numbers of genes that have the combination of motif instances associated with the regions of the diagram. **(C.)** Position weight matrices of the five TF motifs enriched at a less than 5% false discovery rate in the 1000bp regions upstream of significantly changed genes post- infection, taken from the HOCOMOCO database.
(TIF)

S4 Fig Complete Steiner forest network of endothelial cells latently infected with KSHV at 48 hpi. Please refer to legend from Fig 3B for network interpretation.
(TIF)

S5 Fig Steiner forest subnetwork from Metabolism KEGG pathways. Please refer to legend from Fig 3B for network interpretation.
(TIF)

S6 Fig KSHV latently infected endothelial cells induces peroxisome proteins. (A)Flow cytometry of Mock- and KSHV- infected LECs cells harvested at 96 hpi, fixed and stained with PEX3 and MLYCD **(B.)** Geometric mean fold change of KSHV over mock at 96 hpi p < 0.05 student’s t-test. **(C.)** Flow cytometry of Mock- and KSHV- infected TIMECs cells harvested at 96 hpi, fixed and stained with PEX3, PEX19 and MLYCD **(D.)** Geometric mean fold change of KSHV over mock at 96 hpi p < 0.05 student’s t-test. **(E.)** Flow cytometry of Mock- and KSHV- infected hDMVECs cells were harvested at 96 hpi, fixed and stained with PEX3 and MLYCD **(F.)** Geometric mean fold change of KSHV over mock at 96 hpi p < 0.05 student’s t-test.
(TIF)

S7 Fig Distribution of node and edge frequencies in observed and random Steiner forests. We run the Steiner forest algorithm multiple times with the real KSHV protein scores (Observed) and equivalent scores randomly assigned to proteins in the PPI network (Random). Node frequency is the fraction of Observed or Random Steiner forest subnetworks that contain a node, likewise for edges. In general, the nodes and edges that appear in nearly all the Observed subnetworks have a low probability of being included in a Random subnetwork. Very few nodes and no edges lie near the diagonal lines that denote equal frequencies in the Observed and Random subnetworks. The Random subnetworks also contain thousands of nodes and edges that are not relevant to KSHV infection and do not appear in any Observed subnetworks.
(TIF)

S1 Table Complete list of the top KEGG Pathways that overlapped significantly with the predicted Steiner Forest Network. (PDF)

S2 Table Technical replicates of the proteome and phosphoproteome analysis in KSHV infected cells compared to mock infected cells at 48 hours post infection. (XLSX)
