# Supplementary figures and images for: Integrated systems biology analysis of KSHV latent infection reveals viral induction and reliance on peroxisome mediated lipid metabolism
Source: PLoS Pathog. 2017 Mar 3;13(3):e1006256. doi: 10.1371/journal.ppat.1006256 (PMC5352148; doi:10.1371/journal.ppat.1006256)

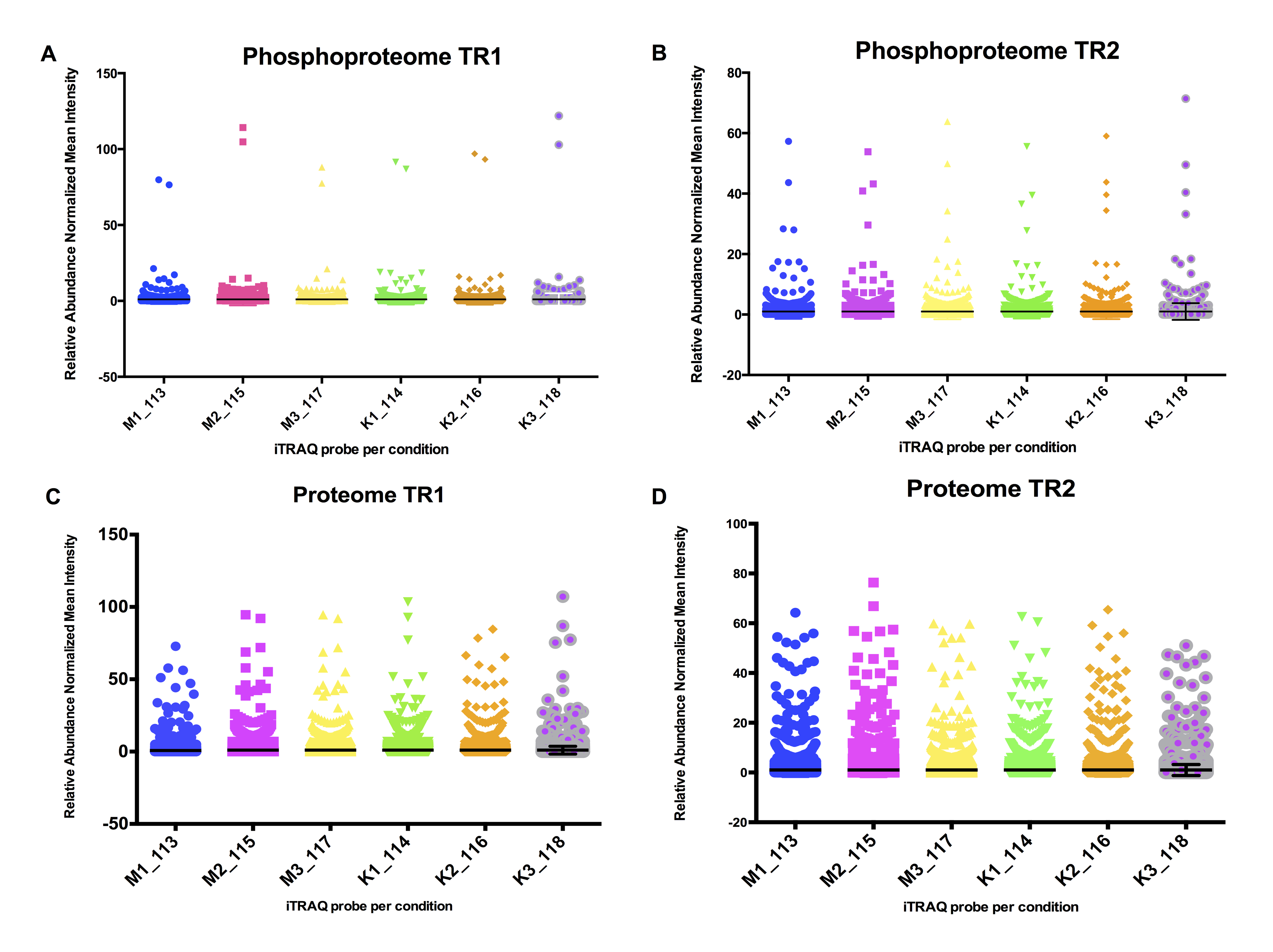

Supplement: S1 Fig — (A.) Phosphoproteome technical replicate (TR) 1. (B.) Phosphoproteome technical replicate (TR) 2. (C.) Proteome technical replicate (TR) 1. (D.) Proteome technical replicate (TR) 2. (TIF) [file ppat.1006256.s001.tif]

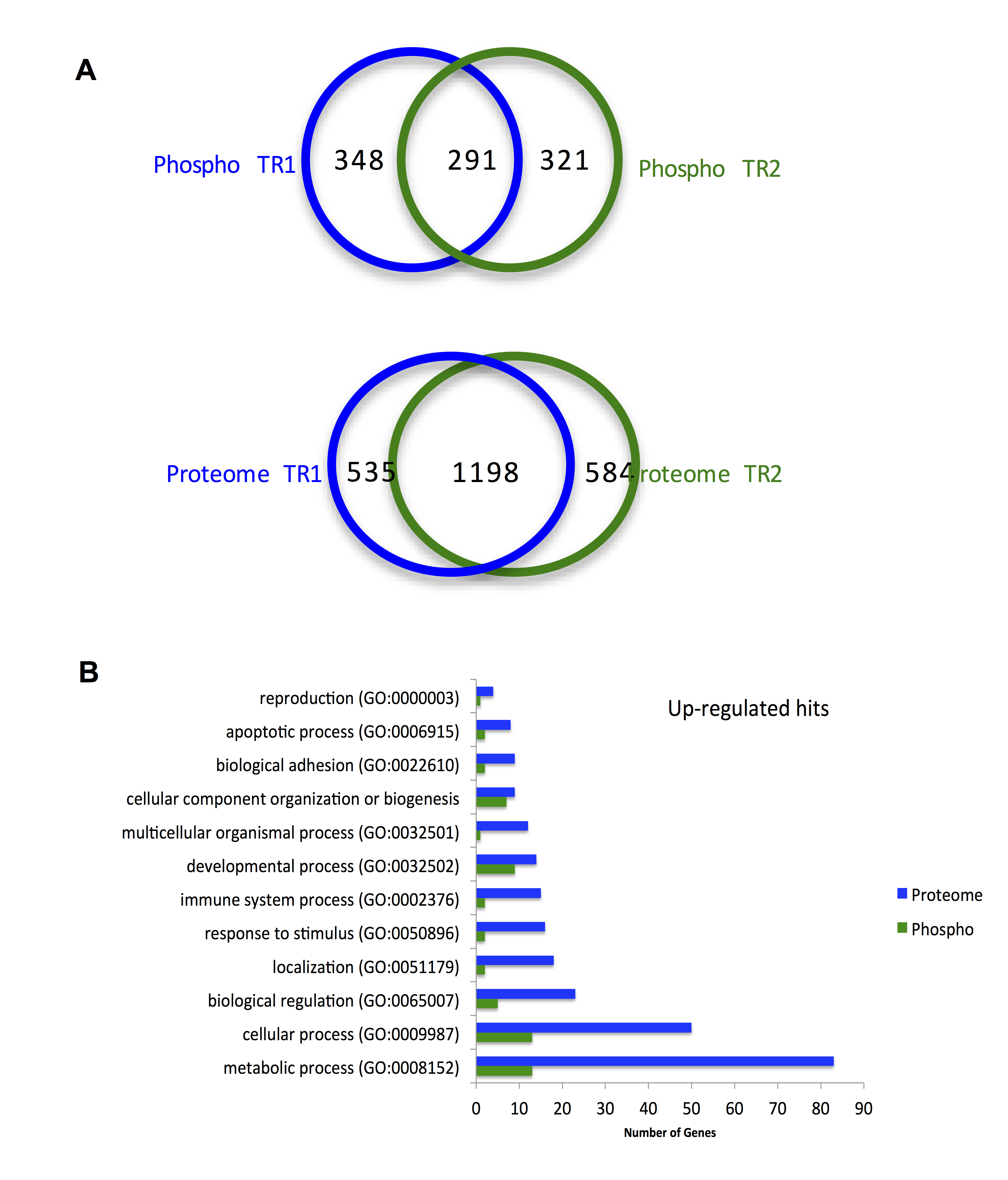

Supplement: S2 Fig — (A.) Venn diagrams of both technical replicate runs from phosphoproteome and proteome. (B.) GO biological process analysis of the upregulated hits from the phosphoproteome and proteome measured proteins. (TIFF) [file ppat.1006256.s002.tiff]

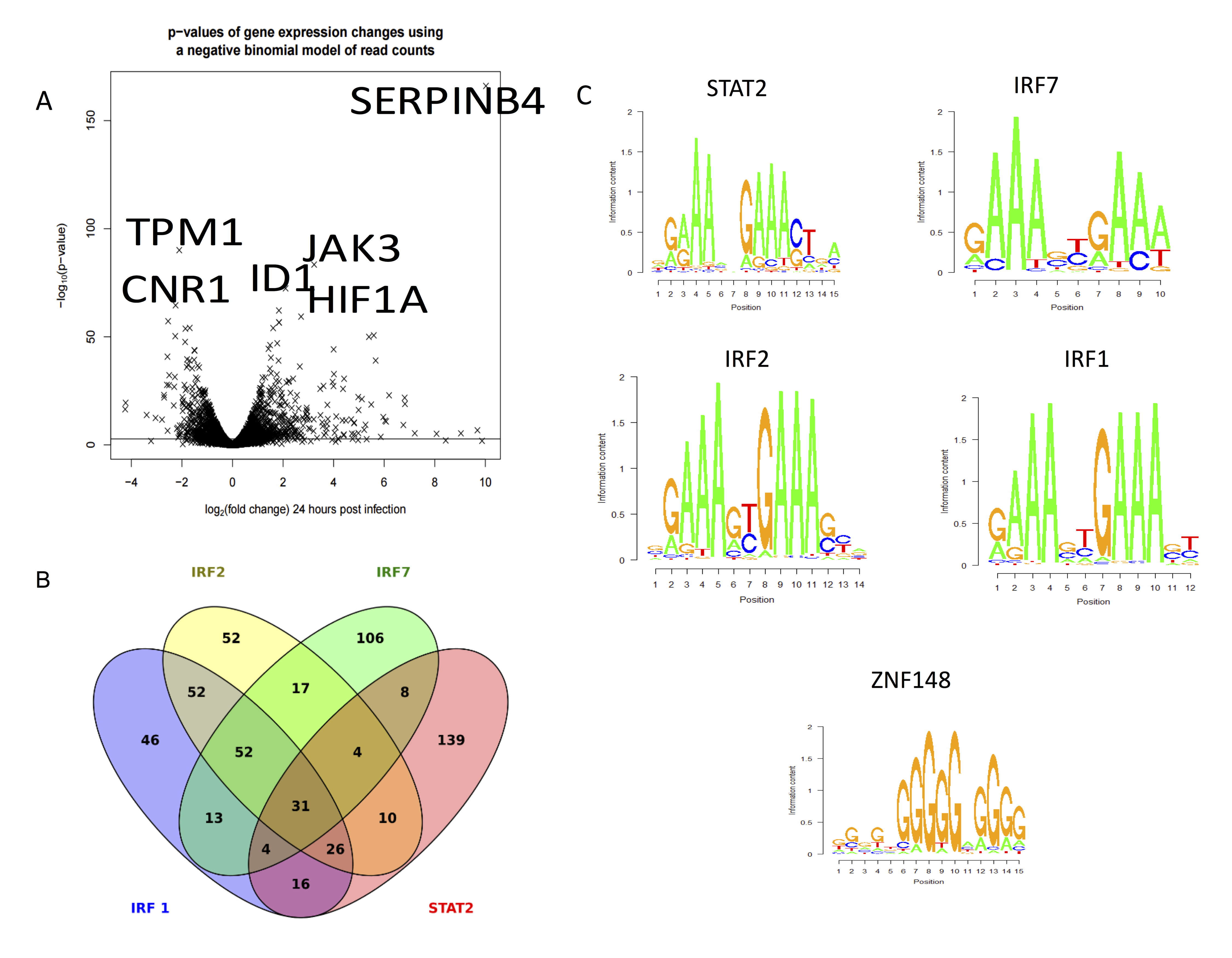

Supplement: S3 Fig — Transcriptomics profiling and motif enrichment comparisons (A.) RNA-seq volcano plot. Highlighting the most highly upregulated and downregulated genes. (B.) Venn diagram of the genes inferred to have motif instances of IRF1, IRF2, STAT2, and IRF7 1000bp upstream of their transcription start site. The numbers show the numbers of genes that have the combination of motif instances associated with the regions of the diagram. (C.) Position weight matrices of the five TF motifs enriched at a less than 5% false discovery rate in the 1000bp regions upstream of significantly changed genes post- infection, taken from the HOCOMOCO database. (TIF) [file ppat.1006256.s003.tif]

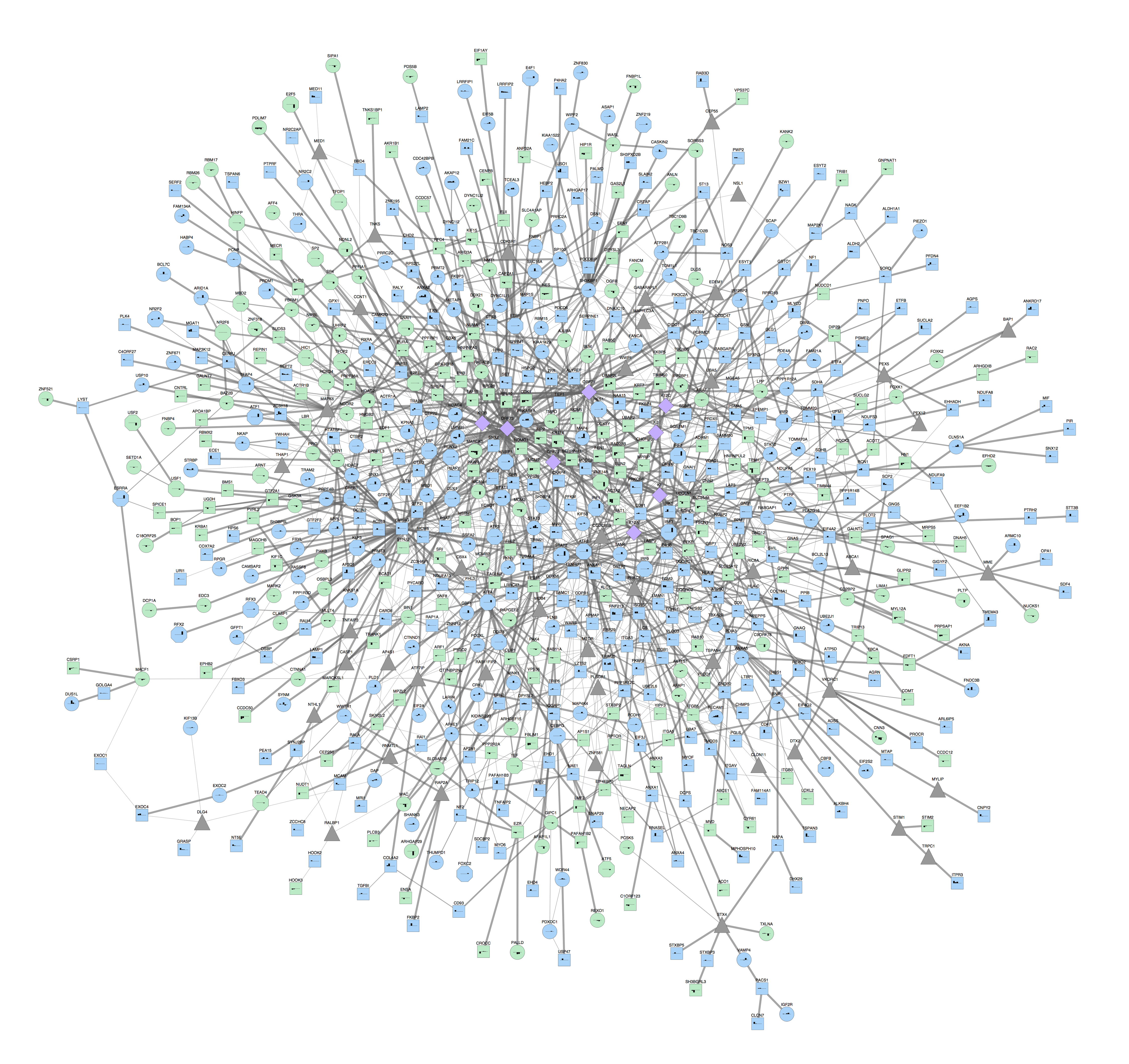

Supplement: S4 Fig — Please refer to legend from Fig 3B for network interpretation. (TIF) [file ppat.1006256.s004.tif]

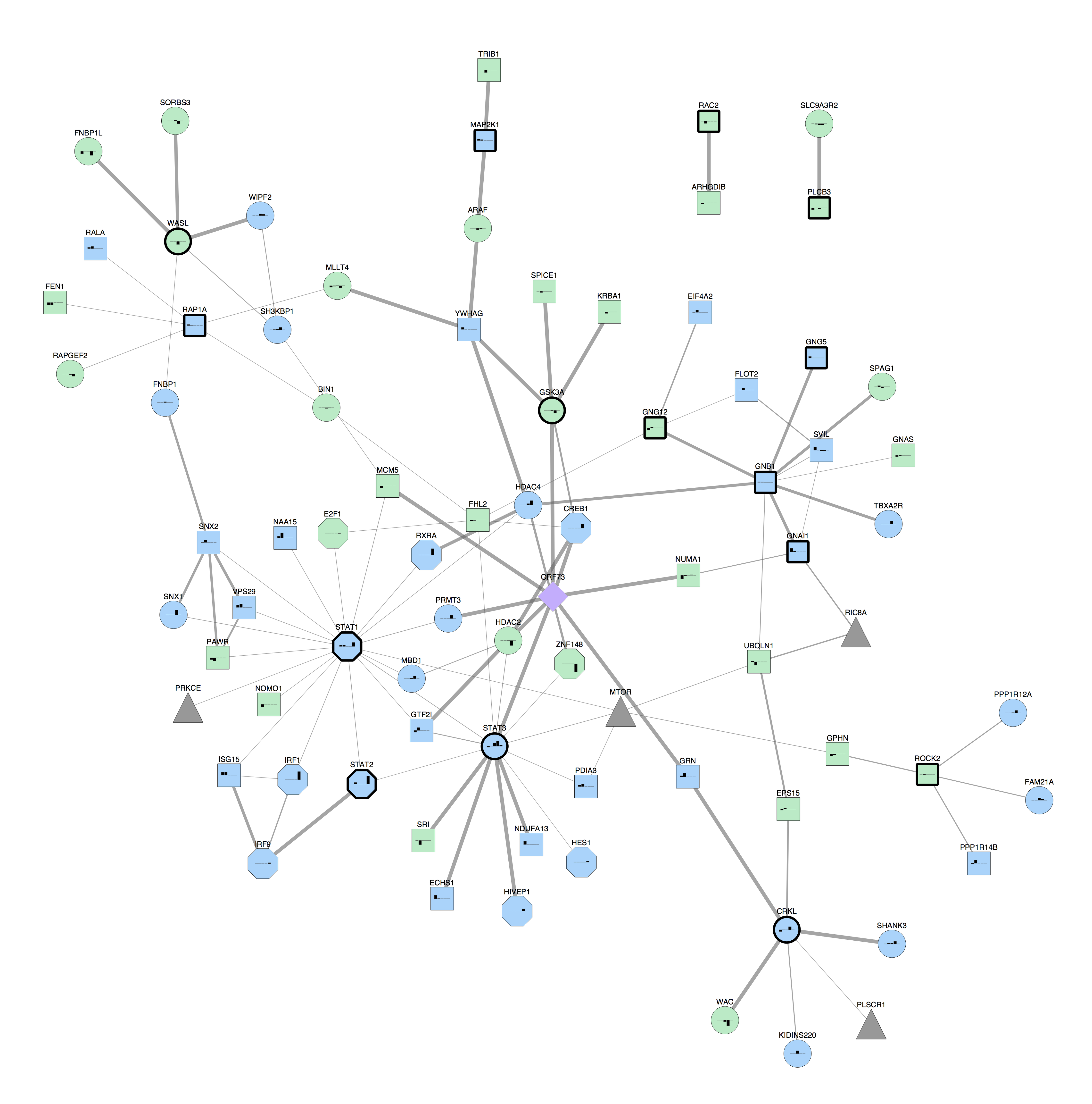

Supplement: S5 Fig — Please refer to legend from Fig 3B for network interpretation. (TIF) [file ppat.1006256.s005.tif]

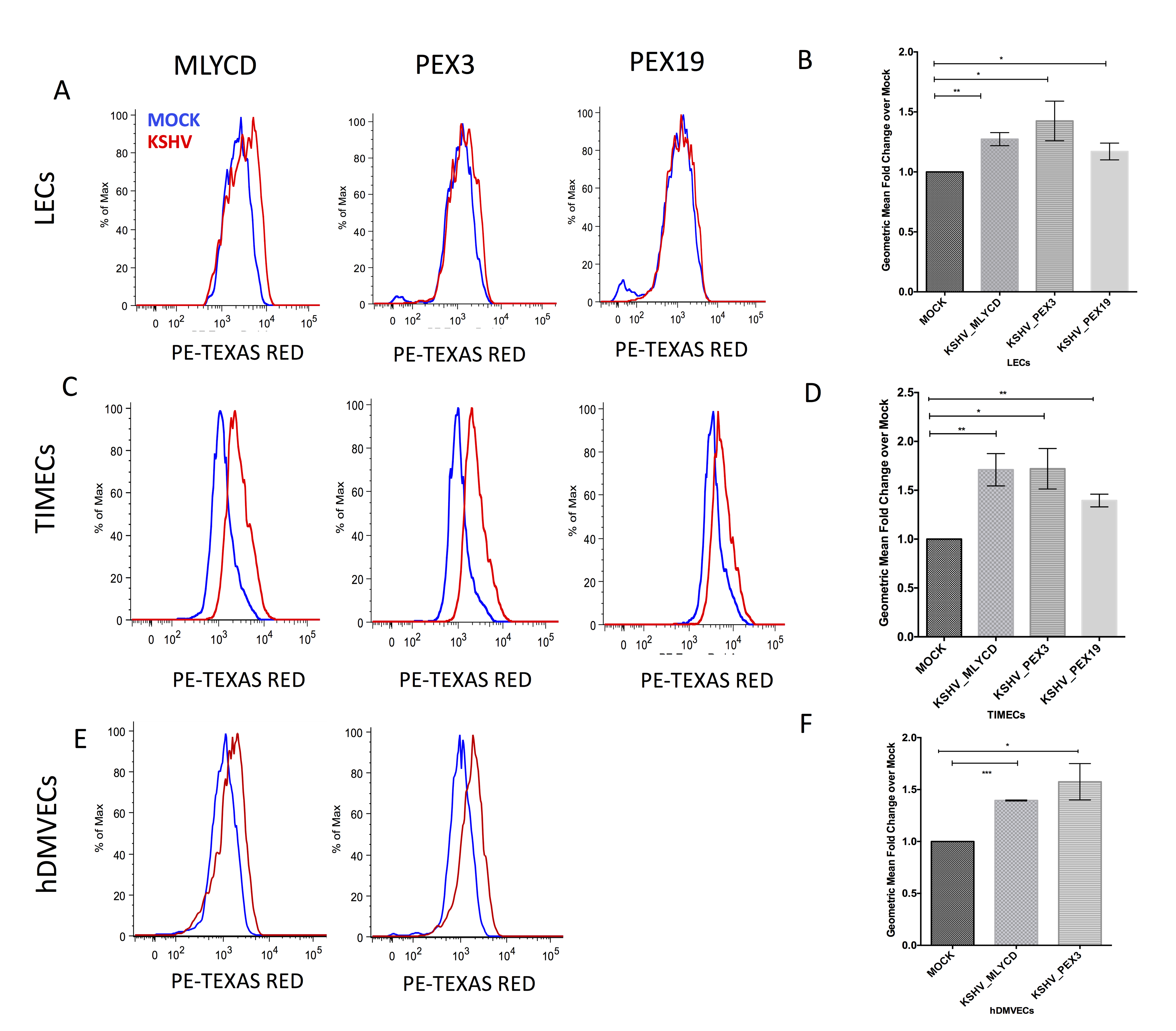

Supplement: S6 Fig — (A)Flow cytometry of Mock- and KSHV- infected LECs cells harvested at 96 hpi, fixed and stained with PEX3 and MLYCD (B.) Geometric mean fold change of KSHV over mock at 96 hpi p < 0.05 student’s t-test. (C.) Flow cytometry of Mock- and KSHV- infected TIMECs cells harvested at 96 hpi, fixed and stained with PEX3, PEX19 and MLYCD (D.) Geometric mean fold change of KSHV over mock at 96 hpi p < 0.05 student’s t-test. (E.) Flow cytometry of Mock- and KSHV- infected hDMVECs cells were harvested at 96 hpi, fixed and stained with PEX3 and MLYCD (F.) Geometric mean fold change of KSHV over mock at 96 hpi p < 0.05 student’s t-test. (TIF) [file ppat.1006256.s006.tif]

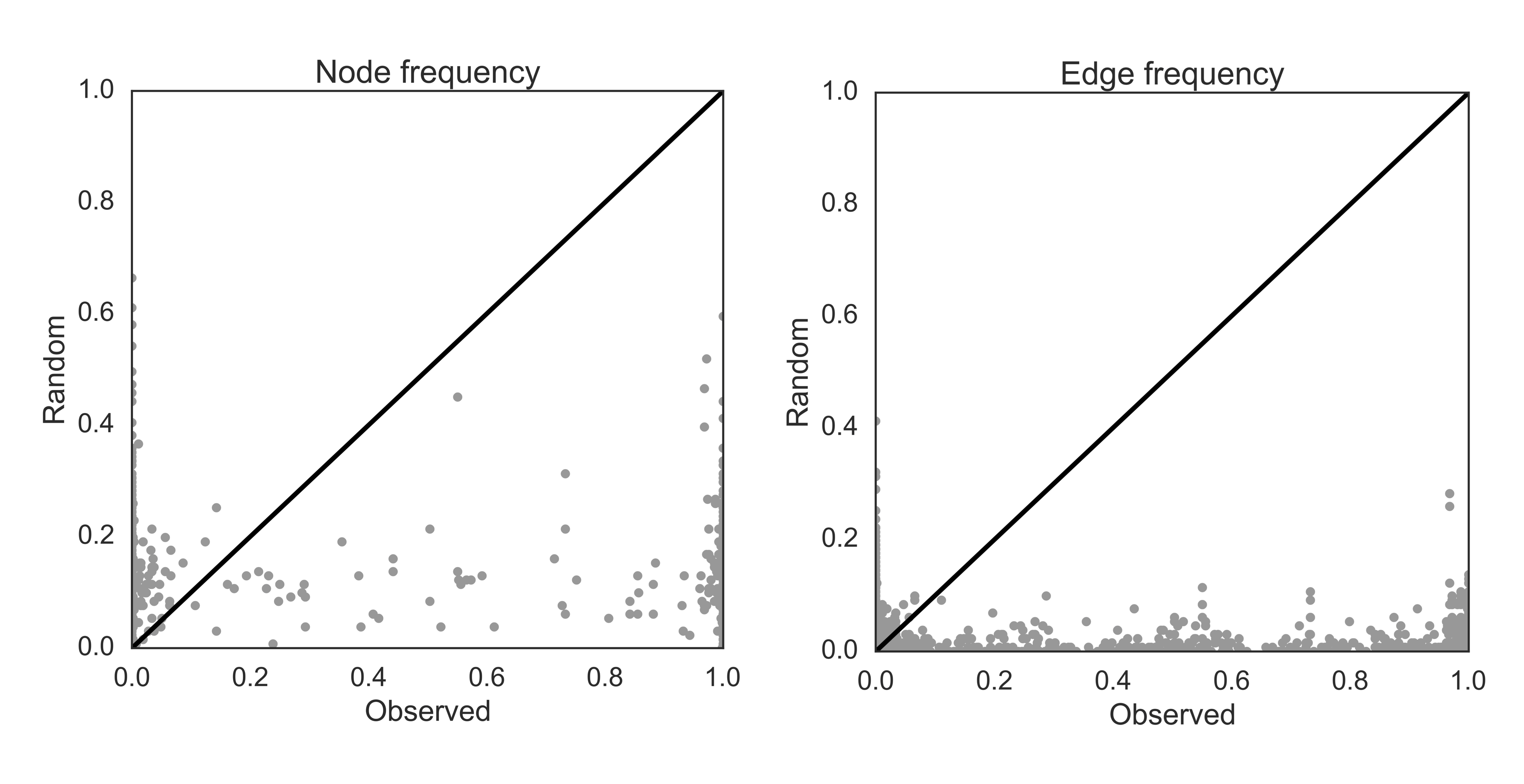

Supplement: S7 Fig — We run the Steiner forest algorithm multiple times with the real KSHV protein scores (Observed) and equivalent scores randomly assigned to proteins in the PPI network (Random). Node frequency is the fraction of Observed or Random Steiner forest subnetworks that contain a node, likewise for edges. In general, the nodes and edges that appear in nearly all the Observed subnetworks have a low probability of being included in a Random subnetwork. Very few nodes and no edges lie near the diagonal lines that denote equal frequencies in the Observed and Random subnetworks. The Random subnetworks also contain thousands of nodes and edges that are not relevant to KSHV infection and do not appear in any Observed subnetworks. (TIF) [file ppat.1006256.s007.tif]
