# Supplementary material for: Integrated systems biology analysis of KSHV latent infection reveals viral induction and reliance on peroxisome mediated lipid metabolism
Source: PLoS Pathog. 2017 Mar 3;13(3):e1006256. doi: 10.1371/journal.ppat.1006256 (PMC5352148; doi:10.1371/journal.ppat.1006256)
Supplement: S1 Table — (PDF) [file ppat.1006256.s008.pdf]

Supplementary Table 1. Complete list of the top KEGG Pathways that overlapped significantly with the predicted Steiner Forest Network.

Pathway name	# Genes	Adjusted p-value
Huntington's disease	24	0.0002
Parkinson's disease	17	0.001
Vasopressin-regulated water reabsorption	10	0.0012
DNA replication	8	0.0061
Alzheimer's disease	18	0.0081
ECM-receptor interaction	12	0.0095
Phagosome	17	0.0104
Endocytosis	20	0.0152
Citrate cycle (TCA cycle)	6	0.0234
Propanoate metabolism	6	0.0243
Leukocyte transendothelial migration	13	0.0243
Hepatitis C	14	0.0243
Protein processing in endoplasmic reticulum	16	0.0336
Long-term potentiation	9	0.0353
Pathways in cancer	26	0.0389
Pancreatic cancer	9	0.0389
Focal adhesion	18	0.0389
Regulation of actin cytoskeleton	19	0.0389
Oxidative phosphorylation	12	0.0389
Adherens junction	9	0.0389
Fc gamma R-mediated phagocytosis	10	0.0578
Lysine degradation	6	0.0578
Peroxisome	9	0.0578
Cell cycle	12	0.0628
RNA transport	13	0.0732
Basal transcription factors	5	0.0732
Dilated cardiomyopathy	9	0.0732
Antigen processing and presentation	8	0.0732
Pancreatic secretion	9	0.0751
Fructose and mannose metabolism	5	0.0751
Vascular smooth muscle contraction	10	0.081
Insulin signaling pathway	12	0.0851
Hypertrophic cardiomyopathy (HCM)	8	0.0858
Long-term depression	7	0.0858
RIG-I-like receptor signaling pathway	7	0.0879
Chemokine signaling pathway	15	0.0963
